# Making a Killer: Selecting the Optimal Natural Killer Cells for Improved Immunotherapies

**DOI:** 10.3389/fimmu.2021.765705

**Published:** 2021-10-27

**Authors:** Samantha A. Barnes, Isabella Trew, Emma de Jong, Bree Foley

**Affiliations:** ^1^Telethon Kids Institute, The University of Western Australia, Nedlands, WA, Australia; ^2^School of Biomedical Sciences, The University of Western Australia, Crawley, WA, Australia

**Keywords:** natural killer cells, cancer immunotherapy, donor selection, cell metabolism, phenotype, epigenetics, transcriptomics

## Abstract

Over the past 20 years natural killer (NK) cell-based immunotherapies have emerged as a safe and effective treatment option for patients with relapsed or refractory leukemia. Unlike T cell-based therapies, NK cells harbor an innate capacity to eliminate malignant cells without prior sensitization and can be adoptively transferred between individuals without the need for extensive HLA matching. A wide variety of therapeutic NK cell sources are currently being investigated clinically, including allogeneic donor-derived NK cells, stem cell-derived NK cells and NK cell lines. However, it is becoming increasingly clear that not all NK cells are endowed with the same antitumor potential. Despite advances in techniques to enhance NK cell cytotoxicity and persistence, the initial identification and utilization of highly functional NK cells remains essential to ensure the future success of adoptive NK cell therapies. Indeed, little consideration has been given to the identification and selection of donors who harbor NK cells with potent antitumor activity. In this regard, there is currently no standard donor selection criteria for adoptive NK cell therapy. Here, we review our current understanding of the factors which govern NK cell functional fate, and propose a paradigm shift away from traditional phenotypic characterization of NK cell subsets towards a functional profile based on molecular and metabolic characteristics. We also discuss previous selection models for NK cell-based immunotherapies and highlight important considerations for the selection of optimal NK cell donors for future adoptive cell therapies.

## Introduction

Natural Killer (NK) cells were first characterized in the 1970s by their ability to detect and eliminate tumor cells without prior antigen sensitization (1). Following observations in the transplantation clinic almost 30 years later, NK cells were identified as one of the first populations to reconstitute following hematopoietic stem cell transplantation (HSCT) and were found to exhibit direct cytotoxicity against malignant cells (2). Indeed, this natural potency against tumor cells has sparked a great deal of interest in exploiting the NK cell platform to treat cancer. Although NK cell-based therapies have not yet achieved the same clinical success as adoptive T cell therapies, early successes in pre-clinical and clinical trials over the past decade have generated enthusiasm for maximizing their therapeutic potential. Several studies have sought to optimize the source from which therapeutic NK cells are derived and the *ex vivo* activation and expansion strategies by which their activity and persistence *in vivo* can be enhanced. However, although the NK cell repertoire is highly heterogeneous both between and within individuals, relatively little attention has been given to the initial selection of NK cells which harbor the greatest antitumor activity. Here, we review the current state of donor selection for peripheral blood NK (pb-NK) cell-based immunotherapies and discuss the factors which drive NK cell effector function along with the challenges associated with identifying highly potent NK cell populations for immunotherapy.

## NK Cell Activation and Antitumor Immunity

NK cells are a cytotoxic subset of innate lymphoid cells (ILCs) with marked potency against malignant cells. NK cells and other ILCs originate from the same bone marrow-derived common lymphoid progenitor cells (CLPs) as B and T lymphocytes (3). Although details of human NK cell development remain largely unknown, bone marrow (BM)-derived CD34^+^CD45RA^+^ CLPs are thought to migrate to various anatomical sites where they subsequently undergo interleukin-15 (IL-15) mediated differentiation along the NK cell lineage (4).

In humans, mature pb-NK cells can be divided into two major functional subsets traditionally characterized based on the relative cell-surface density of the CD56 molecule and expression of the low-affinity IgG Fc region receptor III (FcγRIII; CD16): CD56^bright^CD16^-^ and CD56^dim^CD16^+^. CD56^bright^ cells represent approximately 10% of the pb-NK cell population and primarily act as potent producers of pro-inflammatory cytokines such as interferon gamma (IFNγ) following cytokine stimulation (5). In contrast, CD56^dim^ cells comprise approximately 90% of the pb-NK cell population and produce IFNγ in response to direct interactions with target cells rather than *via* cytokine activation (6). In addition, CD56^dim^ cells are more strongly cytotoxic towards malignant cells than their CD56^bright^ counterparts and harbor high baseline levels of cytotoxic molecules such as perforin and granzyme B (7). The CD56^dim^ population can be further divided into CD57^-^ and CD57^+^ cells, the latter of which represent terminally differentiated NK cells typically considered to harbor the highest cytotoxic potential.

Unlike T and B lymphocytes, NK cell receptors do not undergo somatic rearrangement to generate antigen specificity. Rather, NK cells rely on the stochastic expression of germline-encoded activating and inhibitory receptors, with the complex integration and hierarchy of signals generated through these receptors tightly controlling NK cell function. NK cells express a suite of activating receptors which detect various molecules upregulated by malignant cells. Simultaneous engagement of multiple activating receptors is typically required to overcome an NK cell’s intrinsic activation threshold and trigger effector function (8). A notable exception is CD16, which is the only receptor that can activate NK cells in the absence of other activating signals (9). CD16 is the most potent activating receptor, with crosslinking of CD16 molecules by the Fc region of IgG antibody-opsonized target cells resulting in NK cell activation through a process known as antibody-dependent cell-mediated cytotoxicity (ADCC). Other activating receptors include: the natural cytotoxicity receptors (NCRs) NKp30, NKp44 and NKp46, which directly bind to a wide variety tumor-associated ligands (10); NKG2D, which recognizes the cell-stress induced major histocompatibility complex class I-related molecules MICA and MICB and the UL16-binding proteins (ULBP-1-6) (11); 2B4, which binds to CD48 (12, 13); DNAM-1, which recognizes two protein markers of cellular stress CD112 and CD155 (14); NKp80, which binds to activation-induced C-type lectin (AICL) (15); and the self-associating CD2-like receptor activating cytotoxic cells (CRACC/CD319/CS1/SLAMF7) (16).

NK cells also express a diverse repertoire of inhibitory receptors which recognize human leukocyte antigen (HLA) molecules and regulate self-tolerance to healthy tissues by dominantly inhibiting NK cell activation (17). Two major families of NK cell receptors recognize HLA molecules: the killer immunoglobulin-like receptor (KIR) family and the CD94/NKG2 family of C-type lectin receptors. Up to 15 genes are encoded within the *KIR* locus on chromosome 19 (18), resulting in 14 functional receptors comprising seven inhibitory KIR (-2DL1-2DL3, -2DL5, and -3DL1-3DL3), six activating KIR (-2DS1-2DS5 and -3DS1), and KIR-2DL4 which carries out both activating and inhibitory functions. These KIR genes are highly polymorphic and cluster into haplotypes that differ between individuals, creating at least 40-50 possible *KIR* genotypes and more than 20 haplotypes (18, 19). Haplotypes are divided into two groups characterized by their enrichment for inhibitory (Haplotype A) and activating (Haplotype B) KIRs. Although individual KIR recognize distinct allelic epitopes present in certain HLA-A, HLA-B or HLA-C allotypes, also referred to as KIR ligands, inhibitory KIR have higher avidity for their cognate ligands than activating KIR (20). Similarly, the non-classical HLA molecule, HLA-E, is recognized by both the inhibitory CD94/NKG2A and activating CD94/NKG2C receptors, though the CD94/NKG2A heterodimer binds with higher affinity (21).

Interaction between these major inhibitory receptors and their specific HLA ligand is critical for NK cells to achieve functional maturation through a process known as “licensing” or NK cell education (22). Educated NK cells exhibit the highest reactive potential against target cells that have lost or downregulated HLA expression through a process known as “missing self” recognition, but are susceptible to inhibition by tumor cells that have retained HLA expression (23, 24). Interestingly, as any given inhibitory receptor is present on only a fraction of the NK cell repertoire, both uneducated and educated NK cell populations may coexist within an individual (23). Although the mechanisms underlying NK cell education remain poorly defined, several models have been proposed which debate the relative contributions of inhibitory and activating receptors towards this ongoing process of functional maturation [reviewed (25)].

**Figure 1:**
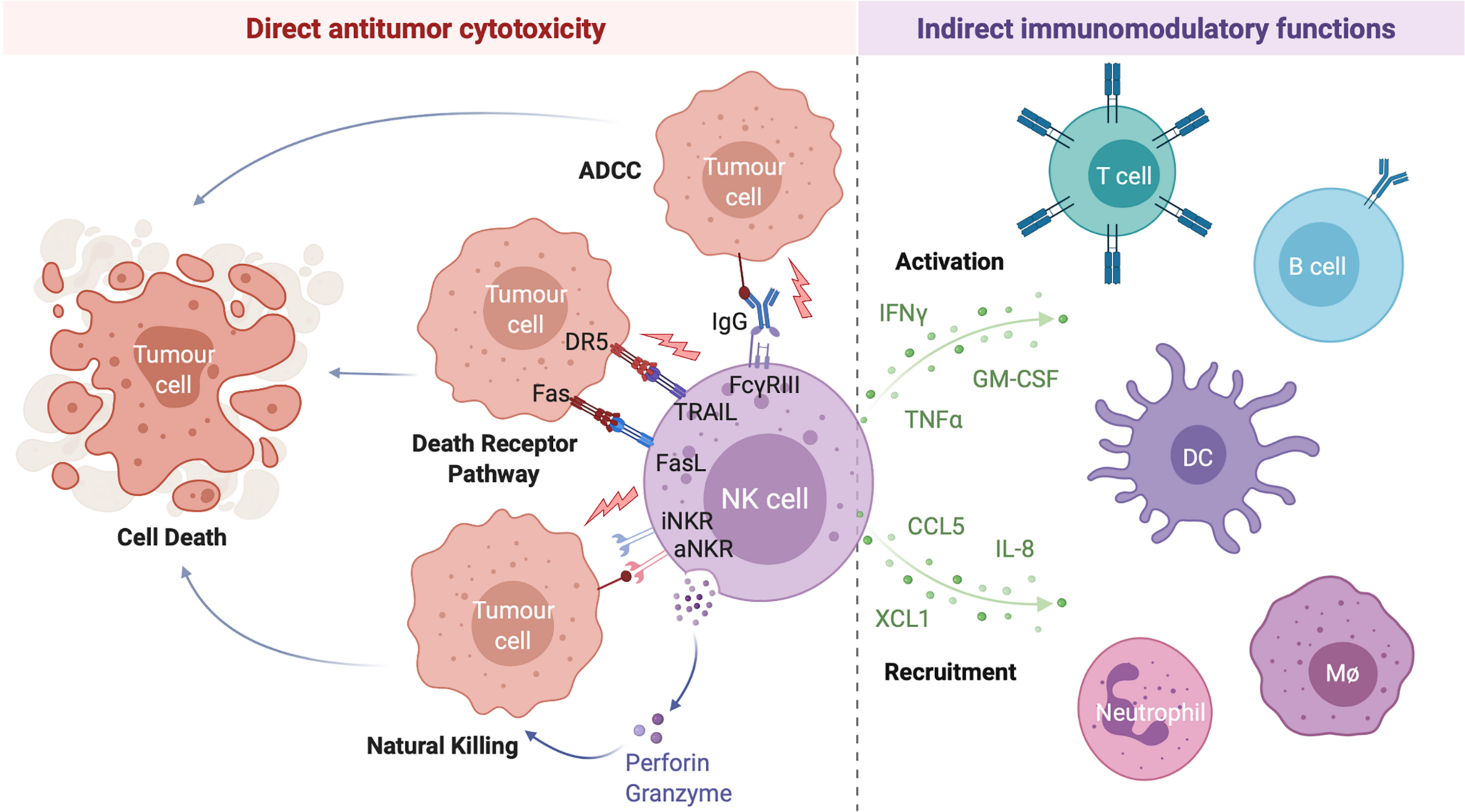
NK Cells Exert a Range of Direct and Indirect Antitumor Effects. NK cell activation is governed by the net balance between signals received through various activating and inhibitory NK cell receptors. When the balance is tipped towards activation, NK cells can directly lyse target cells through release of the preformed cytotoxic granules granzyme B and perforin (natural killing) or by the engagement of target cell death receptors by NK cell-expressed death receptor ligands TRAIL and FasL (death receptor pathway). Antibody opsonized target cells may also be directly lysed through engagement of the NK cell IgG Fc region receptor III (FcγRIII; CD16) in a process of antibody dependent cell-mediated cytotoxicity (ADCC). Activated NK cells are also potent producers of immunomodulatory cytokines (such as interferon gamma (IFNγ), tumor necrosis factor alpha (TNFα), and granulocyte-macrophage colony-stimulating factor (GM-CSF)) and chemokines (including interleukin-8 (IL-8), CCL5, and XCL1) which activate and recruit other immune cells to the tumor microenvironment, indirectly driving a multifaceted antitumor response. Created with (Biorender.com).

Following activation, NK cells carry out a range of antitumor effector functions including the direct lysis of target cells and indirect modulation of both innate and adaptive antitumor immunity through the production of various immunomodulatory cytokines and chemokines (**Figure 1**). Given their potent antitumor activity, relatively low likelihood of severe adverse effects such as graft-*versus*-host disease, and potential for combination with other treatment strategies, NK cell-based therapies have emerged as promising candidates for the treatment of a variety of hematological malignancies and solid tumors (26).

## Development of NK Cell Therapies

The ongoing development of NK cell-based therapies has typically focused on optimizing two major factors: the source from which therapeutic NK cells are derived and the methods by which their activity can be enhanced. NK cells can be obtained from several allogeneic sources including the peripheral blood of related or unrelated donors, umbilical cord blood (UCB), induced pluripotent stem cells (iPSC), and immortalized NK cell lines, with each source harboring intrinsic advantages and disadvantages that must be considered when designing an optimal cellular therapy [reviewed (27, 28)]. Similarly, numerous *ex vivo* enhancements have been developed with the aim of increasing the *in vivo* activity, persistence, and tumor-targeting of the isolated NK cells following infusion. Common strategies include *in vitro* cytokine and feeder cell expansion, cytokine activation in the absence of NK cell expansion, and genetic modification to express a manufactured chimeric antigen receptor (CAR) directed against specific tumor-associated antigens [reviewed (26–28)]. Although several clinical trials are currently evaluating the efficacy of alternative NK cell sources [reviewed (28)], pb-NK cells have remained the most widely utilized source of therapeutic NK cells in clinical trials to date as they are relatively easy to source, have a mature phenotype, and harbor strong cytotoxic activity that can be further enhanced through cytokine stimulation prior to infusion (26). Indeed, pb-NK cells harbor stronger cytolytic activity and greater expression of activating receptors such as CD2 and CD16 than their UCB counterparts (29–31). Furthermore, whilst NK cell lines such as NK-92 represent a robust and renewable source of therapeutic NK cells with strong cytotoxic activity, the requirement for irradiation limits the *in vivo* persistence of the infused cells to a maximum of 48 hours and thus prevents the generation of a long-lasting clinical effect (32). More recently, studies have focused on the development of readily available “off-the-shelf” cellular therapies utilizing unrelated third-party donor-derived pb-NK cells or iPSC-derived NK cells. Indeed, several clinical trials are currently investigating the safety and efficacy of various “off-the-shelf” adoptive NK cell therapy strategies for the treatment of hematological malignancies (**Table 1**).

**Table 1:** Clinical trials of “off-the-shelf” adoptive NK cell therapies for the treatment of hematological malignancies.

Trial Identifier	Therapeutic Agent (source)	Malignancy	Age (years)	Treatment Approach	Study Phase (status)
**NCT04808115**	KDS-1001 (third-party)	CML	All	In combination with TKI therapy	Phase I (not recruiting)
**NCT04848064**	IL-21 expanded “off-the-shelf” NK cells (third-party)	R/R cutaneous T cell lymphoma or T cell leukaemia/lymphoma	18+	In combination with Mogamulizumab	Phase I (not recruiting)
**NCT04632316**	oNKord® (third-party)	AML	18+	In combination with chemotherapy	Phase I/II (recruiting)
**NCT04220684**	mbIL-21 expanded “off-the-shelf” NK cells (third-party)	R/R AML or MDS	1-80	In combination with chemotherapy	Phase I (recruiting)
**NCT04623944**	NKX101 (related donor or third-party)	R/R AML or MDS	18+	In combination with chemotherapy	Phase I (recruiting)
**NCT04310592**	CYNK-001 (third-party iPSC)	AML	18-80	In combination with chemotherapy	Phase I (recruiting)

AML, acute myeloid leukemia; CML, chronic myeloid leukemia; iPSC, induced pluripotent stem cell; NK, natural killer; mbIL-21, membrane-bound interleukin-21; MDS, myelodysplastic syndrome; R/R, relapsed and/or refractory; TKI, tyrosine kinase inhibitors.

## Donor Selection for Improved NK Cell Therapies

Despite the existence of a plethora of strategies to enhance the cytotoxicity and persistence of therapeutic NK cells *in vivo*, the initial selection of highly functional cells with strong innate potency is essential for the widespread success of NK cell therapies. Over the past decade it has become increasingly evident that not all NK cells have the same baseline capacity to eradicate leukaemic cells (33–37). In addition to the functional differences between traditional NK cell subsets (33–35), variability also exists in the functional capacity of NK cells derived from different individuals (36, 37). However, relatively little attention has been given to optimizing the particular subset or donor from which these cells are derived. We have recently reported that resting donor-derived pb-NK cells display marked variability in their capacity to mount an effector response against leukaemic target cells (36). Intriguingly, we identified a pool of donors with strong activity against multiple leukaemic cells, representing ideal candidates for the development of efficacious “off-the-shelf” NK cellular therapies. However, there is currently no standardized criteria by which NK cell donors are selected to improve clinical efficacy. We believe the intrinsic diversity in NK cell activity between individuals is an important consideration when developing and optimizing pb-NK cell-based therapies. Specifically, the selection of donors who harbor NK cells with high baseline anti-tumor activity may provide the opportunity to further improve the success of future NK cell therapies.

### Donor Selection for HSCT

**Figure 2:**
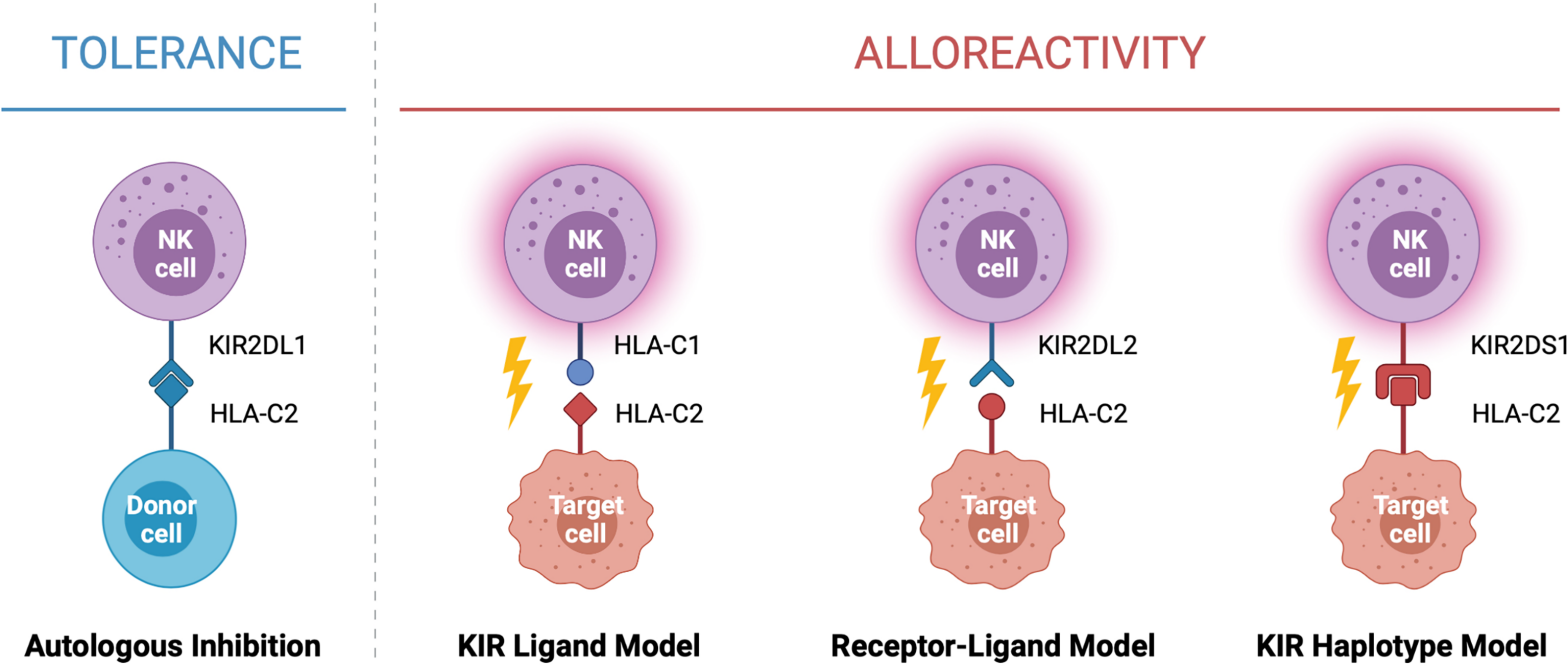
Models of NK Cell Alloreactivity Based on KIR and HLA Expression. Donor NK cells maintain tolerance to self through interactions between inhibitory killer immunoglobulin-like receptors (KIRs) and their cognate human leukocyte antigen (HLA) ligands expressed on healthy autologous cells. In the context of haploidentical HSCT, interactions between donor and recipient KIR and HLA mediate NK cell alloreactivity against target cells. Three models have been described which predict NK cell alloreactivity: mismatch between the donor and recipient HLA (KIR ligand model), mismatch between the donor KIR and recipient HLA (receptor-ligand model), and expression of specific donor KIR haplotypes enriched for activating KIR (KIR haplotype model). When alloreactivity is predicted in the graft-*versus*-host direction, donor NK cells become activated and carry out cytotoxic effector functions against the recipient’s tumor cells. Created with (Biorender.com).

In the context of HSCT, donor selection is critical for preventing graft rejection, graft *versus* host disease and reducing the risk of relapse. When selecting a donor for allogeneic HSCT the goal is to find the closest HLA match to the recipient, typically a sibling or unrelated donor with genetically identical HLA. While this can be difficult to identify for many recipients, a half-matched donor, known as a haploidentical donor, can be found for most individuals. This scenario presents an opportunity for NK cell alloreactivity, in which a mismatch between the donor and patient KIR and/or HLA leads to NK cell activation and results in the elimination of residual leukaemic cells following HSCT (**Figure 2**). This strategy was first exploited by the Perugia group nearly two decades ago (2). In this seminal study, acute myeloid leukaemia (AML) patients receiving haploidentical HSCT experienced enhanced engraftment success, decreased rates of graft rejection, decreased risk of relapse, and increased overall survival compared to those without predicted alloreactivity (2). Strikingly, the five-year overall survival rate for patients receiving HSCT in which NK cell alloreactivity was predicted in the graft-*versus*-host (GvH) direction was 65% compared to 5% in patients without predicted alloreactivity (2). This observation formed the KIR ligand model of donor selection, in which favorable donors were selected by predicting NK cell alloreactivity based on the HLA genotype of the donor and recipient. Ignited by these drastic increases survival, multiple groups used this KIR ligand model of predicted NK cell alloreactivity to investigate its effect on overall survival rates in historic HSCT datasets [reviewed (38)]. Farag and colleagues reported on over 1500 unrelated transplants for AML, chronic myeloid leukaemia (CML), and myelodysplastic syndrome (MDS) and found no association with NK cell alloreactivity and reduced risk of relapse in these diseases (39). In contrast, Hsu and colleagues reported a beneficial effect of NK cell alloreactivity in a cohort of 1770 patients receiving fully ablative T cell replete HSCT for a range of diseases (40). Similar beneficial effects of NK cell alloreactivity were reported in a cohort of over 2000 patients with AML, CML or MDS (41). However, as these observations arose from retrospective analyses of historic datasets spanning a variety of diseases and treatment regimes, it remained difficult to establish the true impact this model of NK cell alloreactivity had on HSCT outcomes.

To enable better prediction of NK cell alloreactivity, Leung and colleagues (42) described a more refined model of donor selection that involved assessing incompatibilities between the recipient’s HLA and the donor’s inhibitory KIR repertoire. This receptor-ligand model of donor selection was suggested to be better at predicting the risk of relapse following HSCT, particularly in patients with lymphoid disease (42). However, using this receptor-ligand model of donor selection, Cook and colleagues reported worse overall survival in transplants between donors carrying the activating *KIR* gene KIR2DS2 and HLA-C2 homozygous recipients when compared to recipients with the HLA-C1 allele (43). This led to more intensive focus on the presence or absence of certain KIR genes and their association with risk of relapse and overall survival. Following analysis of a cohort of over 1400 HSCT recipients, Cooley and colleagues reported that donor *KIR* genotype influenced transplantation outcomes for patients with myeloid but not lymphoid disease (44). Favorable outcomes were observed if the recipient received a transplant from a donor with at least one KIR B haplotype, with the greatest outcomes observed if the donor was homozygous for *KIR* genes within the centromeric region of haplotype B (Cen-B). These observations formed the basis of the KIR haplotype model of donor selection (44). Other studies have also reported similar associations with the Cen-B haplotype, especially with KIR2DS2 [reviewed (38)]. Intriguingly, this contradicts the aforementioned study by Cook et al. which reported a negative association between KIR2DS2 and patient outcomes (43). In a large cohort of over 1200 patients, Venstrom and colleagues reported an association between donor KIR2DS1 (Tel-B gene) and protection against relapse, however this was only observed for donors with HLA-C1 ligands and not those homozygous for HLA-C2 (45). Venstrom et al. also reported reduced recipient mortality with the presence of donor KIR3DS1 (Tel-B gene). Recent studies continue to report associations between activating KIR and disease outcomes, including in children and lymphoid diseases (46–49). Interestingly, similar hierarchical responses have been reported for the inhibitory KIR, KIR3DL1, and its corresponding ligand HLA-Bw4 (50). Indeed, KIR3DL1 and HLA-B combinations resulting in weak or no inhibition towards the recipient’s cells were associated with significantly lower rates of relapse in a study of over 1300 patients with AML (50).

While there doesn’t appear to be a consensus on using KIR haplotypes to select donors, a comprehensive database of *KIR* sequences exists to allow clinicians to assess presence of *KIR* genes and their content to aid in donor selection if desired (51). However, major discrepancies in the literature regarding the association of HLA and/or KIR with beneficial or detrimental outcomes following HSCT makes it difficult to understand the extent to which predicted NK cell alloreactivity actually contributes towards the elimination of leukaemic cells post-transplant (38). Many factors likely contribute to differences reported between studies and transplant centers around the world, including donor source, method of T cell depletion, preparative regimens (fully-ablative or reduced conditioning), and the inclusion and/or type of prophylaxis strategy to mitigate graft-*versus*-host disease. Nevertheless, two main factors contribute to the success of HSCT: the function of the NK cells post-transplant and the ability of these NK cells to target and eliminate tumor cells. If NK cell function is poor and they fail to recognize the tumor, then donor selection based on genetics has little influence on disease outcomes. Thus, a greater appreciation for the underlying functional state of the NK cells should form an important consideration when attempting to describe associations between HSCT strategies and improved patient outcomes. Furthermore, while NK cells are well-known to be the first lymphocyte to reconstitute following transplantation, engrafting NK cells are developmentally immature and exhibit lower effector function compared to healthy donor NK cells (52–57). Interestingly, we demonstrated that CMV reactivation post-HSCT has a significant impact on the reconstituting NK cell repertoire, enhancing NK cell effector function (54). Moreover, presence of CMV-expanded NK cells (now collectively referred to as adaptive NK cells) in HSCT recipients has been correlated with improved outcomes (34). Yet it remains unclear how these adaptive NK cells are involved in better clinical outcomes. Furthermore, additional research is required to better understand the associations between activating KIR and improved outcomes post-HSCT. While KIR2DS1 has been shown to recognize HLA-C2 and mediate alloreactivity against cancer cells (58–61), a direct role for KIR2DS2 is harder to decipher (62). KIR2DS2 can recognize HLA-C in combination with specific peptides (63, 64) and may potentially interact with certain HLA-A alleles (36, 65, 66) or non-HLA ligands (48, 67) suggesting many mechanisms for KIR2DS2^+^ NK cells to target cancer cells post-HSCT. Gaining a better understanding of how associations with activating KIR result in improved responses and this information will allow us to not only better select donors for HSCT, but also to consider additional ways we may be able to exploit these findings to enhance NK cell anti-leukaemic responses.

### Donor Selection for Adoptive Cell Therapy

The success of adoptive NK cell therapy also relies upon alloreactivity between the donor NK cells and the recipient’s tumor cells. In the landmark study conducted by Miller and colleagues, significantly higher rates of complete remission were achieved when graft-*versus*-leukaemia alloreactivity was predicted based on the KIR ligand model, in which 3 out of 4 patients (75%) achieved complete remission compared to 2 out of 15 patients (15%) without predicted alloreactivity (68). However, these findings were not replicated in larger patient cohorts with no reported correlation between complete remission and KIR ligand mismatch (69). Although freshly isolated and activated NK cells have been investigated in several clinical trials to date, the use of *ex vivo* expanded NK cells has become the focus of many ongoing and upcoming trials [reviewed (26)]. However, there is currently no standard criteria by which donors are selected to generate these expanded NK cell therapies. Expanded NK cells display greater expression levels of activating receptors such as NKG2D and NCRs and exhibit significantly greater levels of cytotoxicity against tumor targets compared to resting NK cells (70, 71). Interestingly, KIR/KIR ligand interactions and prior *in vivo* education have been reported to influence NK cell activity following *ex vivo* activation and expansion strategies (72). Specifically, expanded NK cells were found to be more potent when they expressed one or more “licensed” KIR, for which the donor had the corresponding KIR ligand genotype, reflecting the *in vivo* process of education that had occurred prior to isolation and expansion (72). Based on these *in vitro* findings, Wang and colleagues have proposed the licensed receptor-ligand mismatch model of donor selection for adoptive NK cell therapy in which the patient is missing a KIR ligand for which the donor has a licensed KIR (73). Based on the frequency of each HLA genotype in the population, the probability of finding a suitable donor for patients missing at least one KIR ligand is high, requiring a screen of between 3 to 8 unrelated donors (73). However, no suitable donor would exist for patients with all three KIR ligands present. Furthermore, whilst the broad groupings of HLA-C1 and KIR2DL2/3, HLA-C2 and KIR2DL1/S1, and HLA-Bw4 and KIR3DL1 are good indicators of which NK cells may be educated in an individual and thus have the capacity to mediate alloreactivity following transfer, not all ligands bind with the same affinity to their KIR receptor, thus resulting in differing functional potentials. Indeed, early after the identification of the ligands for KIR3DL1 (74), hierarchical responses were described between Bw4 alleles that harbor an isoleucine at position 80 *versus* a threonine at the same position (75). This is further complicated by not all HLA-Bw4 alleles binding to KIR3DL1 as predicted (76) and as at the end of 2020 there are 183 reported alleles of KIR3DL1, some of which differ in their expression and interaction with HLA-Bw4 (50, 77, 78). Similar hierarchical responses have also been identified for KIR2DL1, KIR2DL2 and KIR2DL3 and their respective HLA-C ligands (62, 79–81). These differences in binding affinities have been attributed to differing capacity to educate NK cells and form the basis of the tuning or rheostat model of NK cell education where the level of HLA stimulation influences the functional capacity of the NK cell (82–84). Adding to the complexity is the ability of NKG2A to educate NK cells though its ligand HLA-E, with individuals harboring a methionine at position -21 (-21M) of HLA-B more likely to have NK cells educated strongly through NKG2A (85). This is due to HLA-B alleles with -21M generating peptides that can bind to HLA-E whereas other HLA-B alleles with threonine at -21 cannot. Growing evidence from studies of murine NK cells has also highlighted a potential mechanism of MHC class I (MHC-I)-independent education involving non-MHC-I molecules such as CD48 (2B4 ligand), SLAM family member 6 (SLAM6, self-ligand), C-type lectin-related ligand (Clr-b, NKRP1-B ligand), and poliovirus receptor CD155 (TIGIT ligand) [reviewed (86)]. Collectively, this makes it challenging to rely on classic models of predicated educational status when selecting NK cell donors for enhanced antitumor activity. Furthermore, there are also reports of non-educated NK cells mounting effective responses against cancer and virally infected cells (87–90). A greater understanding of how educated and non-educated NK cells respond within the tumor microenvironment *in vivo* is therefore required to accurately select NK cell donors based on educational status for enhanced antitumor activity.

A distinct advantage of using *ex vivo* expanded NK cells for adoptive cell therapy is the ability to generate large numbers of cells from relatively small starting populations. As such, this process presents the unique opportunity to select for specific NK cell populations which may otherwise represent only a small portion of a donor’s circulating NK cell repertoire. A notable example is FATE-NK100, an NK cell immunotherapy product pharmacologically enriched for NK cells with a CMV-driven adaptive phenotype. Specifically, pb-NK cells are isolated from a related CMV-seropositive donor, depleted of CD3^+^ and CD19^+^ lymphocytes, and cultured *ex vivo* for 7 days in the presence of IL-15 and CHIR99021, a small molecule inhibitor of glycogen synthase kinase 3-beta (GSK3β), to generate the final CD3^-^CD19^-^CD57^+^NKG2C^+^ NK cell product (91). Three phase I clinical trials of FATE-NK100 have been undertaken: DIMENSION for the treatment of advanced solid tumors (NCT03319459; ongoing), APOLLO for the treatment of recurrent ovarian cancer (NCT03213964), and VOYAGE for treatment of relapsed or refractory AML (NCT03081780). In the APOLLO trial FATE-NK100 cells were observed to persist and exhibit enhanced cytotoxic function compared to the patient’s endogenous NK cells for up to 21 days, with clinical benefit reported in three of the nine patients recruited (92, 93). The VOYAGE trial has also reported early success, with all refractory AML patients in dose cohort 2 achieving a morphologic leukaemia free state at day 14 (93). Despite the existence of various other NK cell populations with diverse functional outputs, selection of other subsets for enhanced therapeutic potential remains relatively unexplored. To ensure the continued success of adoptive NK cell therapies, new efforts should seek to identify additional populations of NK cells which harbor high baseline antitumor activity.

## Don’t Judge a Book by Its Cover: Discrepancies Between NK Cell Phenotype and Function

For several decades phenotypic analysis has played an integral role in inferring the identity, maturation state, and functional capacity of NK cell populations. For example, the classic model of NK cell maturation describes a gradual downregulation of CD56 expression and acquisition of CD16 and CD57 expression as pb-NK cells progress from an immunoregulatory CD56^bright^CD16^-^ phenotype towards the cytotoxic CD56^dim^CD16^+^ phenotype, before eventually transitioning into the terminally differentiated CD56^dim^CD16^+^CD57^+^ population with the highest cytotoxic activity. Several other phenotypically and functionally distinct subsets have been described in both healthy and diseased states. For example, infection with cytomegalovirus (CMV) drives the expansion of the CD57^+^NKG2C^+^KIR^+^ adaptive NK cell population with an increased capacity for ADCC (35). In contrast, a population of CD56^-^ NK cells with impaired cytotoxicity and ADCC have been described at low frequencies within healthy individuals but are expanded following both acute (94) and chronic (95, 96) viral infections.

Although phenotyping remains an accessible means by which the heterogeneity of the NK cell repertoire can be explored, increasing evidence suggests these phenotypic classifications of maturity and functional state are inherently flawed. For example, the relative expression level of CD56 does not necessarily inform on maturation state, as CD56^dim^CD16^+^ cells can up-regulate CD56 expression upon cytokine stimulation to become CD56^bright^CD16^+^ (97). Similarly, a proportion of NKG2A^-^ clones have been reported to regain NKG2A expression and CD56^dim^CD57^+^ clones lost CD57 expression following expansion with K562 feeder cells (98). Although typically considered a signature of NK cell functional maturation, KIR expression has also been observed on both CD56^dim^ and CD56^bright^ cells (99). Recent studies have raised further discrepancies in the classic functional roles assigned to the CD56^bright^ and CD56^dim^ populations. Although CD56^dim^CD16^+^ cells are traditionally considered the cytotoxic subset, these cells have also been observed to carry out regulatory functions. Following culture with TGF-B, IL-15, and IL-18, CD56^dim^CD16^+^ pb-NK cells demonstrated reduced cytotoxicity and pro-inflammatory cytokine production, but increased secretion of the immunoregulatory protein VEGF-A (100). Similarly, the traditionally “regulatory” CD56^bright^ NK cell subset is also capable of potent anti-tumor activity. Following priming with IL-15, Wagner and colleagues reported that CD56^bright^CD16^-^ pb-NK cells displayed greater cytokine production, degranulation and killing of leukaemic targets than their CD56^dim^CD16^+^ counterparts (101). A population of highly cytotoxic CD56^superbright^ NK cells have also recently been described following expansion of patient-derived NK cells with K562 feeder cells (102). In this study it was reported that NK cell degranulation, cytotoxicity, and IFNγ production increased alongside increasing expression of CD56. Furthermore, these expanded CD56^superbright^ NK cells were able to eliminate autologous ovarian tumors *in vivo* in patient-derived xenograft models (102). Consideration of other NK cell markers is similarly unable to address these discrepancies between NK cell phenotype and functional output. For example, both regulatory and cytotoxic NK cells can express high levels of activating receptors such as the NCRs and NKG2D, though stimulation through these receptors elicits distinct functional programs in each subset (100, 103, 104). Furthermore, both NK cell populations can express either high or low levels of inhibitory receptors such as NKG2A and KIRs (101, 102, 105, 106). Collectively, these studies suggest there is no specific combination of markers that can consistently distinguish regulatory and cytotoxic NK cell subsets. In a clinically relevant example, traditional phenotypic markers cannot be used to delineate the potency of a donor’s NK cell response against leukaemic cells (36). Indeed, methods to distinguish NK cells with highly potent antitumor activity remain elusive.

The discovery of NK cell populations with a capacity for “memory-like” effector function has also stretched our understanding of how phenotype relates to functional potential. In a unique immunological phenomenon, CMV shapes the phenotypic and functional properties of the NK cell repertoire by driving the expansion of a subset of CD56^dim^NKG2C^+^ adaptive NK cells with memory-like properties [reviewed (107)]. Although predominantly defined by expression of NKG2C, adaptive NK cells are also considered to have a mature phenotype as they typically lack NKG2A expression, express low levels of NKp30 and NKp46, and have high levels of KIR and CD57 expression (34, 108). Functionally, CMV-driven adaptive NK cells are specialized for enhanced ADCC, producing greater levels of IFNγ following activation through CD16 (109). Intriguingly, expansion of this NKG2C^+^ NK cell population is only observed in approximately one-third of CMV seropositive individuals (35). Whilst it has been revealed that the infecting strain of CMV impacts the degree of adaptive NK cell expansion through peptide-specific interactions between the UL40 peptide-HLA-E complex and the activating NKG2C receptor (110), recent findings have challenged the requirement for NKG2C in generating this memory-like functional fate. Indeed, a population of NK cells with a similar memory-like response to CMV has been described in NKG2C^-/-^ individuals (111). Moreover, adaptive NK cell responses have also been reported following CMV reactivation in patients that had received HSCT from NKG2C^-/-^ donors (112). Adaptive NK cells may actually be defined by an array of phenotypes including the loss of all, some, or none of following signaling proteins: FcϵRIγ, Eat-2, and Syk (35, 113, 114). Whilst it is quite possible that other viruses or environmental exposures may similarly impact upon the NK cell repertoire *in vivo*, most observations are confounded by donor CMV seropositivity (115). Furthermore, as many studies have focused solely on this pre-defined phenotypic classification of NKG2C^+^ adaptive NK cells, other markers that may delineate memory-like populations have not yet been identified. Indeed, not all memory-like NK cells display a classically mature phenotype. For example, a population of cytokine-induced memory-like (CIML) NK cells have been described following *in vitro* stimulation with IL-12/IL-15/IL-18 (116, 117). Although this CIML NK cell population is primarily composed of CD56^dim^ cells, the expression of NKG2A, CD25, CD69, CD94, and lack of KIR and CD57 expression suggests a more immature phenotype (116, 117). Strikingly, CIML NK cells display significantly enhanced IFNγ production following cytokine restimulation or target cell activation compared to conventional NK cells, and have since gone on to achieve preliminary success in a phase I clinical trial for the treatment of AML (118). A subset of tissue-resident NK cells with memory-like activity has also recently been described in the context of pregnancy (119). This population of pregnancy-trained decidual NK cells harbored a unique CD56^bright^CD16^-^NKG2C^+^ phenotype and displayed enhanced production of IFNγ and VEGF-A compared to conventional decidual NK cells (119). Taken together, these studies emphasize the vast heterogeneity of memory and memory-like NK cell responses. Moreover, the capacity for these stimuli to shape the functional fate of the NK cell repertoire, and drive a broad spectrum of different phenotypes, highlights the difficulty in assigning functional properties based on phenotypic analysis.

Further discrepancies between NK cell phenotype and function are encountered when attempting to describe dysfunctional NK cell populations. In cancer patients or the setting of chronic viral infection, dysfunctional NK cells are typically characterized by their reduced functional capacity including decreased cytotoxicity in response to target cell stimulation and reduced expression of IFNγ and granzyme B [reviewed (120, 121)]. However, as these are general markers of dysfunction, specific dysfunctional states such as exhaustion, anergy or senescence are difficult to define. Whilst several studies have reported that functional exhaustion of NK cells in tumors or chronic infections is accompanied by the downregulation of activating receptors such as NKG2D, CD16, NCRs, DNAM-1, and 2B4, or the upregulation of markers of T cell exhaustion such as PD-1, TIGIT, TIM-3 and LAG-3, it remains controversial whether NK cells even undergo exhaustion (120). As such, phenotypic markers of NK cell dysfunction remain ill-defined. Indeed, there is currently no established phenotype that can consistently distinguish dysfunctional from functional NK cell populations.

Collectively, these studies demonstrate that there remains no unifying phenotype by which NK cell maturation, functional state, or capacity for memory can be defined, which has important implications for the generation of NK cell products to achieve maximum therapeutic benefit. Indeed, if these classic phenotypes are truly unable to predict NK cell activity, then what other measures can be used to inform on an NK cell’s functional potential?

## Adding Fuel to the Fire: Can Metabolism Inform NK Cell Function?

**Figure 3:**
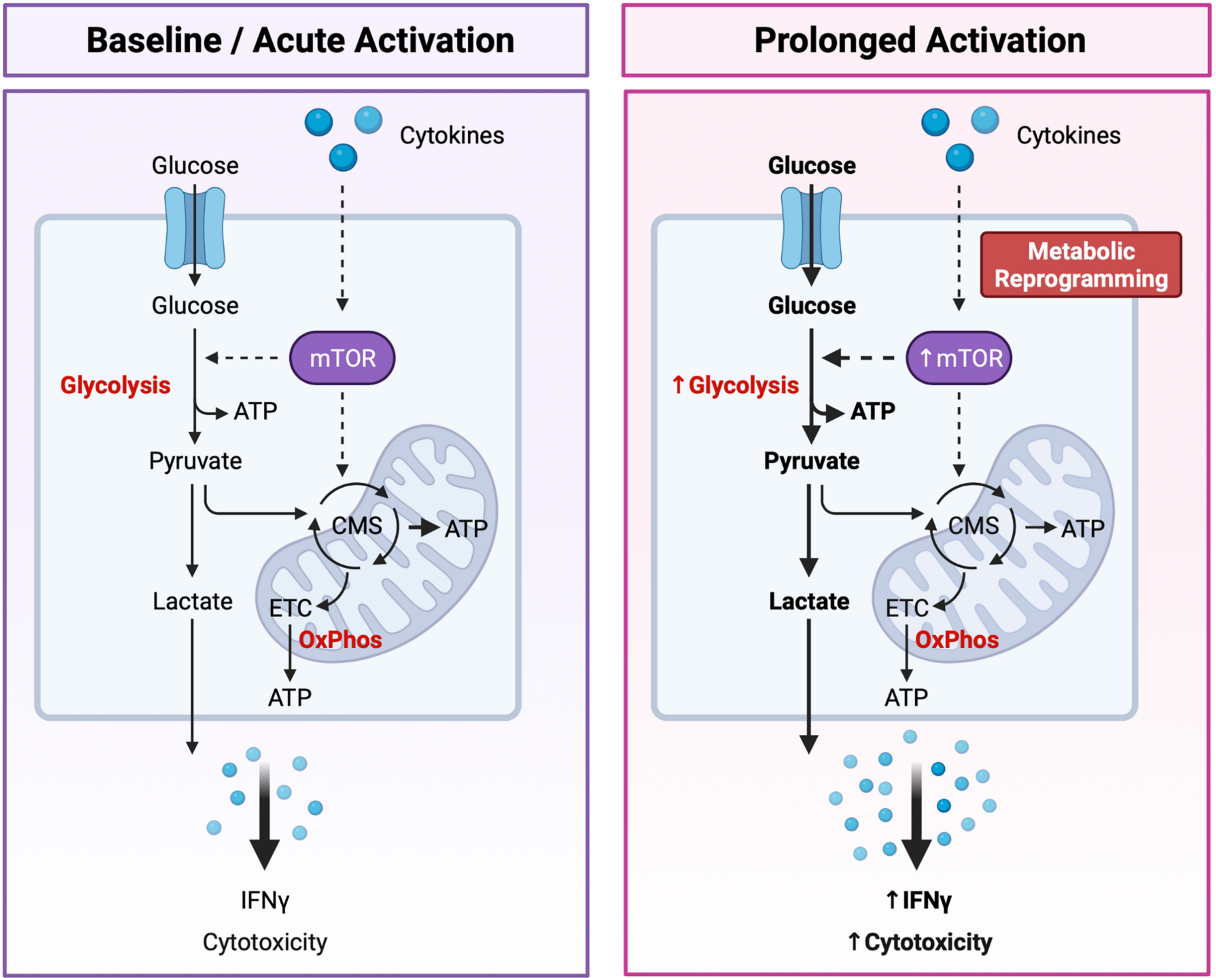
Dynamic Changes in Glucose Metabolism Underly NK Cell Effector Potential. NK cells are primarily fueled by glucose. Following uptake into the cytoplasm, glucose is first converted to pyruvate through glycolysis, generating two molecules of adenosine triphosphate (ATP) per glucose molecule. Pyruvate is then either converted to lactate and expelled from the cell or transported to the mitochondria where it is further metabolized through the citrate malate shuttle (CMS) to fuel the electron transport chain (ETC) and oxidative phosphorylation (OxPhos), driving efficient production of ATP. Basal rates of glycolysis and OxPhos are sufficient to fuel the homeostatic needs and acute effector functions of resting NK cells. Following prolonged cytokine stimulation NK cells experience an increase in the rates of glycolysis and OxPhos to support their increased capacity for IFNγ production and cytotoxic activity. Highly cytotoxic NK cells can also undergo cytokine-induced metabolic reprogramming towards glycolysis through the mechanistic target of rapamycin (mTOR), a master regulator of cellular metabolism. An increased capacity for glucose-driven metabolism and more robust activation of the mTOR pathway identifies NK cells with the greatest cytotoxic potential. Created with (Biorender.com).

It is now apparent that cellular metabolism is not only a means by which cells generate energy and biochemical precursors required for homeostasis, but it is intrinsically tied to immune cell function. Recent studies have reported that immunometabolism plays a critical role in regulating NK cell development, education, activation, and memory response [reviewed (122, 123)]. As metabolism plays such an integral role in dictating NK cell biology, a new paradigm has emerged in which distinct “metabolic fingerprints” underpin NK cell functional fate (122). Specifically, differences in the capacity for and regulation of glucose-driven metabolic pathways may identify NK cells with enhanced cytotoxic potential (**Figure 3**).

Dynamic changes in the glucose-driven metabolic pathways glycolysis and oxidative phosphorylation (OxPhos) coincide with NK cell development and effector function. In mice, developing NK cells utilize both glycolysis and OxPhos to fuel the energy-intensive process of proliferation (124), whereas mature NK cells are considered metabolically quiescent at steady-state and preferentially use OxPhos to meet their homeostatic needs (125). Although less is known about the metabolic requirements of developing NK cells in humans, mature pb-NK cells also demonstrate low rates of glycolysis and OxPhos at resting state (126). Interestingly, metabolic differences have been reported between the CD56^bright^ and CD56^dim^ NK cell compartments. Resting CD56^dim^ NK cells have a greater mitochondrial mass and demonstrate higher rates of glycolysis and OxPhos compared to the CD56^bright^ subset (127). CD56^dim^NKG2C^+^ adaptive NK cells isolated from CMV seropositive individuals also exhibit an increased capacity for glycolysis and OxPhos compared to donor-matched CD56^dim^NKG2C^-^ canonical NK cells (128). However, basal levels of these pathways were comparable between adaptive and canonical NK cells, suggesting that adaptive NK cells may have a greater capacity to upregulate these metabolic pathways following activation (128). As NK cells are poised to respond rapidly following activation it is perhaps unsurprising that the rates of glycolysis and OxPhos remain unchanged during short-term cytokine stimulation (4 hours with IL-15 and/or IL-12 and/or IL-18) or receptor ligation (6 hours with anti-NK1.1 or anti-Ly49D) (125). However, inhibition of either pathway was shown to significantly impair IFNγ production, especially following receptor ligation. Taken together these findings suggest that the low basal metabolic rate of resting NK cells is sufficient to fuel acute NK cell effector responses. Interestingly, several studies have also reported that prolonged NK cell activation drives robust changes in cellular metabolism. Indeed, overnight stimulation with cytokines significantly increases the rates of glycolysis and OxPhos in both human and murine NK cells (124, 126, 129, 130). Accumulating evidence suggests that this increased level of glucose-driven metabolic fitness drives enhanced NK cell cytotoxicity. For example, NK cells stimulated with IL-15 for 3-5 days demonstrate higher rates and overall capacity for glycolysis and OxPhos and exhibit greater levels of IFNγ production in response to receptor-mediated activation compared to those that receive short-term stimulation of 4-24 hours (124, 125). Furthermore, this prolonged IL-15 stimulation eliminated the metabolic requirement for NK cell activation, with IFNγ production sustained following OxPhos inhibition (125). Metabolic reprogramming may also underpin the enhanced cytotoxic capacity of educated NK cells. Schafer and colleagues reported that following activation and expansion using IL-21-expressing K562 feeder cells, educated NK cells were metabolically reprogrammed towards glycolysis and mitochondrial-dependent glutaminolysis to support their increased cytolytic activity, whereas the uneducated subset relied solely upon OxPhos (131). In addition, metabolic fitness plays an important role in governing NK cell effector function within the tumor microenvironment (TME) [reviewed (132, 133)]. Limited nutrient availability within the TME may restrict NK cell metabolism, thus impairing effector function (134–136). Accumulation of tumor-derived metabolites within the TME has been reported to dampen NK cell activity through impairing key metabolic pathways. For example, adenosine has been shown to inhibit the metabolic activity of human NK cells by inhibiting OxPhos and reducing their glycolytic capacity (137) and uptake of lactic acid by NK cells leads to intracellular acidification and impaired energy production (138). More recently, Poznanski and colleagues reported that NK cell dysfunction within the TME is due to suppression of glucose-driven metabolic pathways *via* lipid peroxidation-associated oxidative stress (139). Strikingly, expanded NK cells reprogrammed towards complete metabolic substrate flexibility demonstrated greater metabolic fitness and enhanced antitumor activity against ovarian tumors *in vivo* (139). These findings suggest that an increased capacity for glucose-driven metabolism and high metabolic fitness may identify NK cells with enhanced cytotoxic activity and thus greater therapeutic potential.

Tight regulation of the mechanistic target of rapamycin (mTOR) is also critical for NK cell development and activation. mTOR is a highly evolutionarily conserved serine/threonine kinase comprised of two distinct complexes: mTOR complex 1 (mTORC1) and mTOR complex 2 (mTORC2). Together, these complexes act as master regulators of cellular metabolism and integrate signals for nutrient availability, growth, and activation to adjust the rates of glycolytic metabolism and biosynthesis accordingly (140). Several studies have reported that mTOR signaling plays a crucial role in the early stages of NK cell development (124, 141, 142). For example, IL-15-induced activation of the mTOR pathway is required for E4BP4 expression in developing BM-NK cells, which in turn promotes transcription of Eomes and drives commitment towards the NK cell lineage (141). More recently, crosstalk between mTORC1 and mTORC2 was found to promote NK cell maturation through controlling the expression of transcription factors Tbx21 and Eomes in a cooperative and non-redundant manner (143). Interestingly, mTORC1 and mTORC2 were also reported to regulate NK cell metabolism and anti-tumor activity in opposing ways. NK cells from mTORC2 deficient mice displayed greater cytolytic activity and increased metabolic rate compared to their wild-type counterparts, whilst cytotoxicity and cellular metabolism were significantly diminished in mTORC1 deficient NK cells (143). Other studies have also demonstrated that mTOR plays a critical role in controlling NK cell activation in both mice and humans, with IL-15 induced activation of the mTOR pathway required for priming of cytotoxicity in the periphery (124, 144). Marçais and colleagues reported that mTOR activity downstream of the IL-15 receptor increased granzyme B expression in both murine and human NK cells, whereas inhibition of mTOR by rapamycin abrogated NK cell cytotoxicity (124). Interestingly, IL-15 stimulation has been reported to activate the PI3K/Akt/mTOR pathway more robustly in CD56^bright^ NK cells compared to the CD56^dim^ population, corresponding with their potent increase in cytotoxicity following prolonged stimulation (101). Transforming growth factor-β (TGF-β), a major immuno-suppressive cytokine well-known for its role in inhibiting NK cell cytotoxicity, has also been reported to directly suppress NK cell activation through inhibition of mTORC1 and through mTORC1-independent inhibition of mitochondrial metabolism (130, 145). Indeed, *in vitro* treatment with TGF-β reduced the metabolic activity, cytotoxicity, and abundance of various NK cell receptors in both murine and human NK cells (130). Furthermore, deletion of the TGF-β receptor subunit TGF-βRII in murine NK cells enhanced mTOR activity and NK cell cytotoxicity *in vivo* (130). Together, these findings highlight the importance of mTOR activity in regulating NK cell antitumor function both *in vitro* and in the tumor microenvironment. Differences in mTOR activity, or in the relative activity of the mTORC1 and mTORC2 pathways, may therefore be useful in delineating NK cell cytotoxic potential. Moreover, boosting the metabolic activity of NK cells through targeting the mTOR pathway may be an effective strategy for enhancing the antitumor activity of NK cell-based therapies and thus warrants further investigation.

It is now apparent that metabolism plays an essential role in dictating NK cell functional fate. As metabolism is intrinsically tied to NK cell survival and antitumor activity, the potential to manipulate NK cells *ex vivo* for enhanced metabolic fitness holds promise for enhancing the efficacy of NK cell-based therapies. Indeed, several strategies have been proposed to genetically or pharmacologically “rewire” NK cell metabolism to promote *in vivo* longevity, improve tumor recognition, sustain antitumor function, increase trafficking to the tumor site, and protect the adoptively transferred NK cells from the tumor microenvironment itself (146). As more tools than ever before are now available to interrogate NK cell metabolism [reviewed (147)], future investigations should also seek to define specific “metabolic fingerprints” which can be used to identify NK cells with the highest therapeutic potential. Although our understanding of NK cell metabolism is currently in its infancy, a deeper appreciation of the interplay between metabolism and molecular regulators of NK cell functional fate, as discussed below, holds promise for unlocking the full potential of NK cell therapies.

## Programmed to Kill: Molecular Determinants of NK Cell Function

The fields of transcriptomics and epigenomics are rapidly advancing. Recent breakthroughs in the development of high-resolution and high-throughput sequencing technologies have enabled researchers to explore the transcriptional and epigenetic landscape of NK cells in more depth than ever before. However, our understanding of the molecular regulation of NK cell functional fate is still in its infancy. Relatively little is known about the molecular pathways and regulatory programs that underly NK cell development, effector function, and memory response. As a central goal of genetic and epigenetic studies involves understanding the factors that drive individual variation, a deeper understanding of NK cell biology at the molecular level may also aid in identifying optimal donors for NK cell immunotherapy.

The development of high-resolution transcriptomic analyses such as RNA sequencing (RNA-seq) and single cell RNA-seq (scRNA-seq) has provided researchers with unprecedented insight into the developmental and functional plasticity within the NK cell compartment. A particular interest has arisen in unravelling the developmental trajectory of human NK cells. Based on phenotypic analyses, the current model of NK cell differentiation describes a linear relationship between the immature CD56^bright^ and terminally differentiated CD57^+^ NK cell populations. However, the well-established loss of CD56^bright^ but not CD56^dim^ NK cells in GATA2-deficient individuals challenges this current dogma of NK cell development (148). Using scRNA-seq to analyze NK-lineage cells derived from a donor with the GATA2^T354M^ mutation, Yang et al. confirmed the loss of CD56^bright^ cells in this donor due in part to a higher rate of apoptosis compared to GATA2-sufficient cells (149). Furthermore, whilst the heterogeneity of this donor’s NK cell repertoire was mostly intact compared to healthy controls, defects in steady-state activation were also observed (149). Although the developmental trajectory of these GATA2-deficient NK cells remains unclear, transcriptomic analyses of healthy donor-derived NK cells supports the linear model of differentiation, suggesting that the CD56^bright^ subset is a precursor to the CD56^dim^ population with CD57^+^ cells representing the terminal stage of NK cell differentiation (149, 150). Interestingly, analysis of CD56^bright^CD16^-^ and CD56^dim^CD16^+^ NK cell populations derived from various tissues suggests that NK cell developmental and functional fate is shaped by the tissue site from which they are derived (151). Indeed, tissue-specific transcriptional patterns of maturation, distribution, and function were largely maintained across donor age, sex and CMV serostatus (151). Yang et al. have also described the shared presence of five distinct NK cell clusters across the BM and peripheral blood derived from healthy donors (149). Interestingly, two of these five clusters (“Mature NK” and “Terminal NK”) were predicted to form the CD56^dim^CD57^+^ population together, suggesting that this classically terminally differentiated NK cell subset is not as homogenous as previously thought (149). An additional three novel subsets of pb-NK cells have been described using scRNA-seq, including type I IFN-responding CD56^neg^ NK cells, CIML NK cells, and a small population of NK cells with reduced ribosomal expression, decreased OxPhos and markers of cellular activation (152). Heterogeneity within the CD56^dim^CD16^+^CD57^-^ subset was also observed, with two distinct subpopulations distinguished based on the relative abundance of chemokine mRNA and frequency of KIR-like receptor expression (152). In addition to this high level of variation within an individual’s NK cell repertoire, several studies have also reported the presence of strong donor phenotypes in scRNA-seq datasets (149, 153, 154), likely reflecting the unique genetic profile and immunological history of each donor. As these donor phenotypes are present under physiological conditions, exploration of the transcriptomic differences between donors may uncover an even deeper level of NK cell functional heterogeneity than previously described by phenotypic analysis alone. Indeed, several studies have reported the presence of adaptive NK cell clusters in CMV seropositive individuals (149, 153), and an “inflamed” NK cell cluster specific to one BM donor (149). However, the development of new annotation tools and more robust sequencing technologies may be required to interrogate the full heterogeneity of the NK cell repertoire between healthy donors. More recently, Crinier and colleagues have reported strong donor phenotypes in BM-NK cells derived from AML patients at diagnosis (153). Remarkably, the extent of transcriptomic heterogeneity between AML patients was so high that traditional annotation tools were unable to identify conserved NK cell subsets, even when considering patients at the same classification of AML (153). Interestingly, the overall transcriptomic profile of BM-NK cells from AML patients was enriched for genes involved in cytokine and type I IFN signaling pathways, whereas healthy BM-NK cells displayed a transcriptomic profile enriched for genes involved in NK cell cytotoxicity (153). These findings highlight the strong donor-specific effects AML carries out on the endogenous NK cell repertoire. However, the relationship between these distinct transcriptomic profiles and patient outcomes has not yet been elucidated. Similarly, the extent to which other hematological malignancies impact on the endogenous NK cell compartment, or on adoptively transferred NK cells, remains unexplored. Indeed, further investigation is needed to fully appreciate the role transcriptomic regulation plays in controlling NK cell activity in health and disease.

Epigenetic alterations are reversible and heritable changes to the genome that do not alter the DNA sequence itself, but have profound impacts on gene expression, cell phenotype, and functional fate. Broadly, NK cell development and effector function is regulated through various epigenetic alterations including DNA methylation, histone modification, transcription factor (TF) changes, and microRNA (miRNA) expression [reviewed (155)]. NK cells undergo profound epigenetic remodeling throughout their development. For example, gradual demethylation of gene promoters at the *KIR* and *IFNG* loci during NK cell differentiation corresponds with acquisition of KIR expression (156) and the ability to produce IFNγ (157), respectively. The dynamic interplay between chromatin accessibility and gene expression levels throughout NK cell development has recently been described (158). Using the Assay for Transposase-Accessible Chromatin using sequencing (ATAC-seq) to assess changes in genome-wide chromatin accessibility levels of different developmental stages of *in vitro*-derived NK cells, Li and colleagues revealed the presence of two distinct TF clusters that regulate NK cell differentiation (158). Additionally, two novel TFs were identified (FOSL2 and EGR2) and found to be essential for controlling NK cell maturation and function (158). However, as this study utilized an *in vitro* model of NK cell differentiation it is unclear how these findings will translate to the natural *in vivo* process of NK cell development. Indeed, Li and colleagues have reported differences in chromatin accessibility between these *in vitro*-derived NK cells and their naturally occurring counterparts (158). Nevertheless, the identification and characterization of TFs and transcriptional regulatory networks involved in NK cell differentiation presents an opportunity to artificially drive NK cell functional fate along a desired pathway *in vitro*. Indeed, pharmacological inhibition of GSK3 kinase during *ex vivo* NK cell expansion with IL-15 is currently used to drive late-stage maturation and enhanced effector function of FATE-NK100 cells through upregulating the expression of TFs such as T-BET, ZEB2, and BLIMP-1 (91). Chromatin dynamics also play an important role in regulating the function of the mature NK cell repertoire. For example, target cell recognition drives alterations in the NK cell histone methylation state that correspond with changes in gene expression levels (159). Interestingly, small-molecule compounds targeting H3K4 and H3K27 methyltransferases and demethylases were able to mimic these activation-induced histone modification states in the NK92MI cell line, inducing significantly greater levels of degranulation (UNC1999) and expression of IFNγ and TNFα (OG-L002 and MM102) compared to untreated controls (159). Entinostat, a histone deacetylase inhibitor, also modulates NK cell effector function through modifying chromatin accessibility (160). Mechanistically, treatment with entinostat was reported to increase chromatin accessibility of the *IFIT1* gene promoter region, driving the epigenetic upregulation of the IFIT1-mediated IRF1, STAT4 and STING pathways, and resulting in increased NK cell cytotoxicity against tumor targets (160). Although entinostat was found to enhance NK cell cytotoxicity, it remains unclear how other epigenetic modifying drugs (several of which are currently in clinical trials for the treatment of various cancers) may directly or indirectly impact upon NK cell function (155). Furthermore, the breadth of other exposures which can imprint upon the NK cell epigenetic landscape and thus modulate NK cell activity is currently unknown.

Perhaps the most notable example of an environmental exposure driving epigenetic remodeling of the NK cell repertoire is the CMV-driven expansion of adaptive NK cells. The unique phenotypic and functional characteristics of this “memory-like” adaptive NK cell population corresponds with epigenetic imprinting at the regulatory regions of genes encoding IFNγ, FcϵRIγ, EAT-2, and PLZF (35, 113, 161). For example, demethylation of the *IFNG* locus increases the accessibility of the CNS1 region and drives the characteristic increase in IFNγ expression displayed by adaptive NK cells (161). Conversely, hypermethylation of the *FCER1G* and *SH2D1B* (encoding EAT-2) loci corresponds with the reduced expression of these signaling proteins (35). Hypermethylation of an intronic region of the *ZBTB16* locus (encoding PLZF) was also observed, corresponding with a striking 77% downregulation of this transcript in adaptive NK cells compared to conventional NK cell populations (35). Demethylation of the *IFNG* locus at CNS1 has also been reported in NKG2C^-/-^ adaptive NK cells, further highlighting the crucial role epigenetic remodeling plays in driving this unique functional state. Although described only in the context of CMV to date, it remains possible that a broader range of exposures to other viral, bacterial, or even eukaryotic pathogens may also contribute towards a memory-like functional fate (162). However, as many studies have focused solely on known adaptive NK cell phenotypes, distinct functional or epigenetic profiles driven by other environmental stimuli have not been identified. Indeed, it remains unclear how a lifetime of exposures may imprint upon the epigenetic landscape of an individual’s NK cell repertoire. It is also unclear whether *ex vivo* expansion methods or the tumor microenvironment itself may drive epigenetic changes in adoptively transferred NK cells, potentially affecting their persistence and antitumor activity *in vivo*. Nevertheless, as epigenetic alterations are both reversible and druggable, identification of the specific epigenetic signatures underlying enhanced antitumor immunity will yield novel targets that can be exploited to further improve NK cell-based therapies. More broadly, a deeper appreciation of the epigenetic determinants of NK cell functional potential may also aid in identifying and selecting optimal NK cell populations or donors for immunotherapy.

Transcriptomic and epigenomic analyses have uncovered a greater level of heterogeneity, both within and between the NK cell repertoires of different individuals, than previously observed by phenotype alone. As emerging evidence continues to highlight the high level of complexity and plasticity within the NK cell compartment, new studies into the molecular regulation of NK cell functional fate hold great promise for revolutionizing the field of NK cell immunotherapy. Indeed, a comprehensive understanding of the transcriptional and epigenetic programs underlying enhanced NK cell activity or longevity *in vivo* may reveal a plethora of molecular phenotypes and targets that can be exploited to improve future NK cell-based therapies.

## Conclusion and Perspectives

NK cells have tremendous potential to revolutionize the field of cancer immunotherapy. Coupled with their innate potency against cancer and ability to be transferred between donors and patients without mediating severe adverse effects, NK cells have emerged as ideal candidates for the development of readily available “off-the-shelf” therapies. Whilst a range of NK cell sources and *ex vivo* manipulation strategies have been extensively investigated over the past two decades, there remains no standard criteria by which NK cells with enhanced therapeutic potential can be identified and selected for immunotherapy. Indeed, it is now apparent that NK cells displaying similar functions can express a wide variety of phenotypic markers, and individual NK cells within a defined phenotypic population can fulfil a range of distinct functional roles. As accumulating evidence continues to expose discrepancies between the NK cell phenotype and functional output, there is a need to develop new strategies by which NK cell donors or populations with enhanced antitumor potency can be identified.

Advancements in the fields immunometabolism, transcriptomics, and epigenomics have led to an exciting new era for NK cell research, highlighting a deeper level of complexity and plasticity within the NK cell compartment than previously described. Recent studies have leveraged these multi-omics technologies to describe novel determinants of enhanced NK cell activity, including increased rates of glycolytic metabolism, greater metabolic fitness, and epigenetic remodeling towards a “poised” effector state. However, it remains unclear how these metabolic and molecular “fingerprints” can be used to select NK cell donors or populations for greater antitumor activity *in vivo*. Whilst several measures of metabolic fitness and mTOR activity can be assessed simultaneously using flow cytometric analyses [reviewed (147)], efficient profiling of NK cells based on epigenomic or transcriptomic signatures remains unachievable. However, these unbiased approaches are indispensable for gaining a greater appreciation for the heterogeneity in the NK cell repertoire both within and between donors. A comprehensive understanding of how these molecular regulatory programs interact with cellular metabolism and drive NK cell functional fate will aid in developing new strategies for profiling NK cells based on functional potential. Furthermore, a deeper knowledge of these important regulatory pathways will uncover new targets that can be exploited to enhance the efficacy of future NK cell-based therapies.

## Author Contributions

SB, IT, EJ, and BF designed, wrote, and edited the manuscript. All authors contributed to the article and approved the submitted version.

## Funding

The preparation of this review article received no external funding. This work was supported by the Australian Government Research Training Program Scholarship at The University of Western Australia (scholarship to SB), and the Cancer Council Western Australia and the Western Australia Department of Health (research funding to BF).

## Conflict of Interest

The authors declare that the research was conducted in the absence of any commercial or financial relationships that could be construed as a potential conflict of interest.

## Publisher’s Note

All claims expressed in this article are solely those of the authors and do not necessarily represent those of their affiliated organizations, or those of the publisher, the editors and the reviewers. Any product that may be evaluated in this article, or claim that may be made by its manufacturer, is not guaranteed or endorsed by the publisher.
